# AL amyloidosis with non-amyloid forming monoclonal immunoglobulin deposition; a case mimicking AHL amyloidosis

**DOI:** 10.1186/s12882-018-1050-y

**Published:** 2018-11-22

**Authors:** Shun Manabe, Chihiro Iwasaki, Michiyasu Hatano, Fuyuki Kametani, Masahide Yazaki, Kosaku Nitta, Michio Nagata

**Affiliations:** 1 Kidney and Vascular Pathology, Faculty of Medicine, University of Tsukuba, 1-1-1 Tennodai, Tsukuba, Ibaraki, 305-8575 Japan; 2 Department of Nephrology, Yokohama Rosai Hospital, Yokohama, Kanagawa Japan; 3 Department of Medicine, Kidney Center, Tokyo Women’s Medical University, Tokyo, Japan; 4 Department of Dementia and Higher Brain Function, Tokyo Metropolitan Institute of Medical Science, Tokyo, Japan; 5 Institute for Biomedical Sciences, Interdisciplinary Cluster for Cutting Edge Research, Shinshu University, Nagano, Japan

**Keywords:** Immunoglobulin light-chain amyloidosis, Immunoglobulin heavy-and-light chain-amyloidosis, Amyloid purification, Laser microdissection, Mass spectrometry

## Abstract

**Background:**

Immunoglobulin heavy-and-light-chain amyloidosis (AHL amyloidosis) is a newly established disease entity where both the immunoglobulin heavy-chain and light-chain compose amyloid fibrils. The immunoglobulins responsible for the amyloid fibrils are generally identified by immunostaining and/or laser microdissection-liquid chromatography-tandem mass spectrometry (LMD-LC-MS/MS). However, both techniques do not biochemically differentiate immunoglobulins that formed amyloid fibrils from non-responsible immunoglobulins.

**Case presentation:**

We herein report a case of 67-year-old female patient with renal amyloidosis due to lymphoplasmacytic lymphoma secreting monoclonal immunoglobulin M (IgM)-kappa. Renal immunostaining monotypically positive for IgM-kappa and LMD-LC-MS/MS identification of mu heavy-chain and kappa light-chain were consistent with the diagnosis of AHL amyloidosis. In order to confirm that both the immunoglobulin heavy-chain and light-chain were forming amyloid fibrils, we performed LC-MS/MS of renal amyloid fibrils isolated by the traditional amyloid purification method. The additional LC-MS/MS identified kappa light-chain only without any heavy-chain component. These results were suggestive that amyloid fibrils were composed by kappa light-chain only and that the mu heavy-chain identified by immunostaining and LMD-LC-MS/MS was derived from the non-specific co-deposition of monoclonal IgM-kappa.

**Conclusion:**

The case was AL amyloidosis with non-amyloid forming monoclonal immunoglobulin deposition. While immunostaining and LMD-LC-MS/MS are irreplaceable techniques to classify amyloidosis, confident exclusion of the present condition should be required to diagnose AHL amyloidosis.

## Background

Amyloidosis is a group of diseases characterised by deposition of insoluble fibrils derived from various proteins [1]. Immunoglobulin related amyloidosis (Ig-amyloidosis) is one of the most prevalent renal amyloidosis and is classified into immunoglobulin light-chain (AL) amyloidosis, immunoglobulin heavy-chain (AH) amyloidosis, and, the recently established disease entity, immunoglobulin heavy-and-light-chain (AHL) amyloidosis [1]. Most of the renal Ig-amyloidosis is AL amyloidosis, whereas AH and AHL amyloidosis are rare entities [2, 3].

The classification of renal Ig-amyloidosis is generally performed by immunostaining of immunoglobulin light-chains and heavy-chains following positive Congo red stain [1]. Recently, laser microdissection (LMD) of glomerular amyloid depositions combined with liquid chromatography-tandem mass spectrometry (LC-MS/MS) has enabled more accurate classification [1]. In large-scale studies classifying renal amyloidosis by immunostaining and LMD-LC-MS/MS [2, 3], the amyloidosis with monotypic immunoglobulin light-chain and heavy-chain deposition were described as AHL amyloidosis. Therefore, at present, amyloidosis with monotypic immunoglobulin light-chain and heavy-chain deposition, with greater than or equal heavy-chain amount, is diagnosed as AHL amyloidosis [3]. However, immunostaining and LMD-LC-MS/MS do not biochemically differentiate immunoglobulins that formed amyloid fibrils from non-amyloid, just deposited, immunoglobulins [4, 5]. At the same time, the intact immunoglobulins could co-deposit with amyloid fibrils [4–6] and be identified by immunostaining [4, 6] and LMD-LC-MS/MS [4, 5].

We herein report a case of renal Ig-amyloidosis due to lymphoplasmacytic lymphoma, with immunostaining and LMD-LC-MS/MS results consistent with previously reported AHL amyloidosis [2, 3]. We attempted to confirm that both the immunoglobulin light-chain and heavy-chain were forming amyloid fibrils by LC-MS/MS of renal amyloid fibrils isolated by traditional amyloid purification method [7]. The additional LC-MS/MS only identified immunoglobulin light-chains as the component of amyloid fibrils. This case demonstrated “AL amyloidosis with non-amyloid forming monoclonal immunoglobulin deposition” that might be diagnosed as AHL amyloidosis by immunostaining and LMD-LC-MS/MS.

## Case report

### Clinical history and laboratory results

A 67-year-old Japanese woman, taking daily oral cyclophosphamide against lymphoplasmacytic lymphoma for 4 years, was referred to the nephrology department because of recently developed nephrotic syndrome. The patient showed bilateral leg edema and weight gain. Laboratory data showed preserved kidney function (serum creatinine, 0.61 mg/dL; estimated glomerular filtration rate, 73.7 ml/min/1.73m^2^) and hypoalbuminemia (2.2 g/dL). The urinalysis was negative for occult blood and revealed a urinary total protein-to-creatinine ratio (UPCR) of 9.7 g/gCr. Serum immunoglobulin levels were IgG 466 mg/dL, IgA 74 mg/dL, and IgM 2789 mg/dL, serum free light-chain levels were kappa-light chain 24.4 mg/L and lambda-light chain 15.8 mg/L (kappa-to-lambda serum free light-chain ratio of 1.54) and plasma electrophoresis revealed IgM-kappa monoclonal gammopathy. Renal biopsy was performed under suspicion of monoclonal gammopathy of renal significance [1].

### Renal biopsy findings and clinical follow-up

**Fig. 1:**
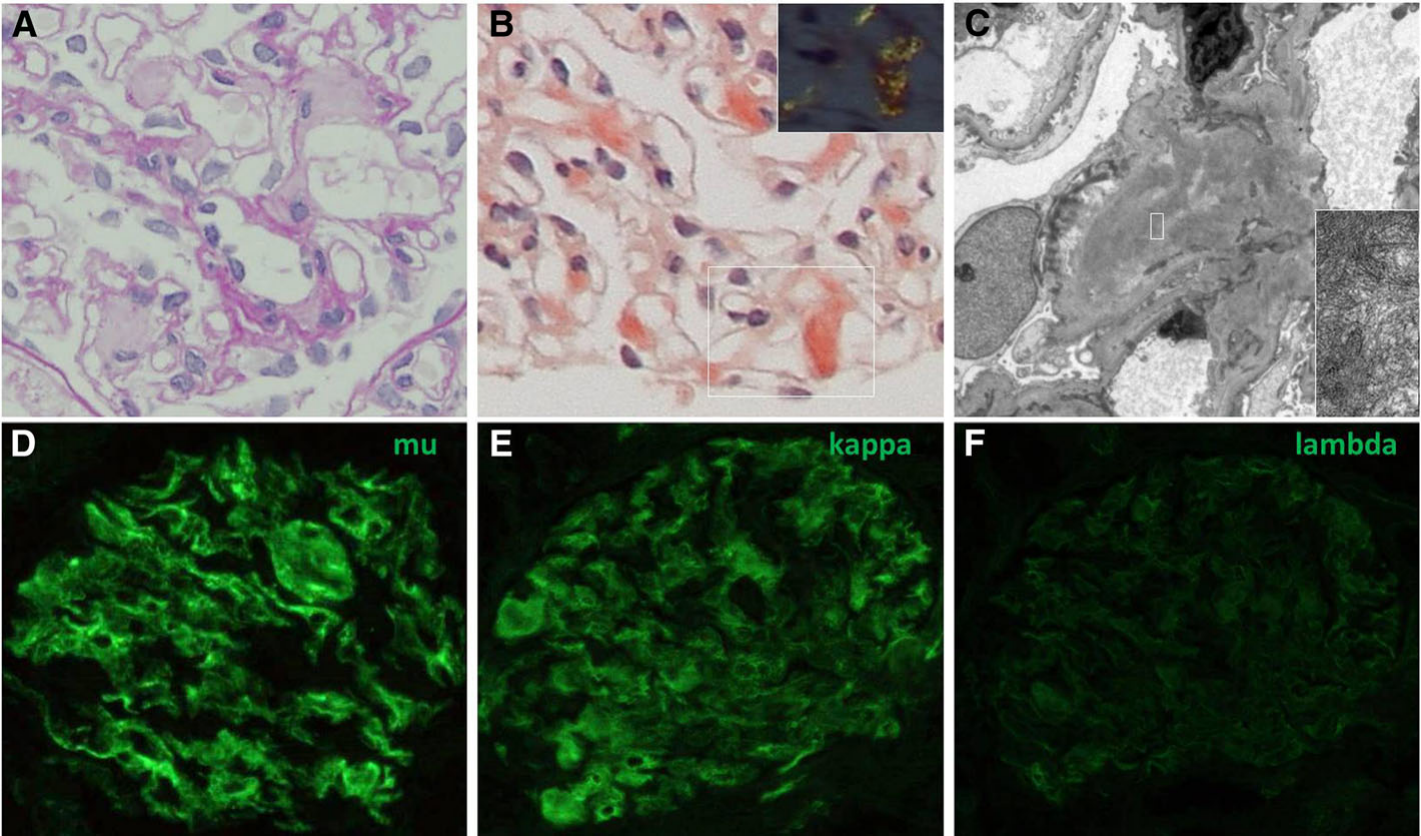
Renal biopsy findings consistent with IgM-kappa type AHL amyloidosis. **a** Glomeruli with mesangial widening consisting amorphous deposition (× 100, periodic acid-Schiff stain), (**b**) Glomeruli with mesangial Congo red positive deposition (× 100), (**c**) Electron micrograph with mesangial organized deposition with fibrillary structure of 8 to 12 nm in diameter (× 1500, × 20000), (**d-f**) Immunofluorescent staining positive for mu heavy-chain (**d**) and kappa light-chain (**e**) and negative for lambda light-chain (F)

The renal biopsy specimens contained 12 glomeruli without proliferative features, and the glomeruli revealed slight mesangial widening with amorphous deposits (Fig. 1a). Congo-red staining was positive in the mesangial area (Fig. 1b) and electron microscopic analysis showed non-branched fibrils (Fig. 1c) indicating renal amyloidosis. Electron microscopic analysis was negative for granular and amorphous deposits on the endothelial aspect of the glomerular basement membrane and outer aspect of the tubular basement membrane. Immunofluorescence staining was equally positive for IgM (mu heavy-chain, Fig. 1d) and kappa light-chains (Fig. 1e) and was negative for linear staining of tubular basement membrane. The immunostaining for the other immunoglobulins (Fig. 1f), complements, serum amyloid A, transthyretin, and fibrinogen yielded negative results. Therefore, IgM-kappa type renal AHL amyloidosis due to lymphoplasmacytic lymphoma was suspected [1]. Smears and flow cytometry of bone marrow aspirate were consistent with previous diagnosis of lymphoplasmacytic lymphoma. After the diagnosis, weekly oral fludarabine was initiated. The proteinuria and hypoalbuminemia gradually improved to UPCR of 2.5 g/gCr and serum albumin level of 3.6 g/dL in one-and-a-half years.

### LC-MS/MS analyses

**Fig. 2:**
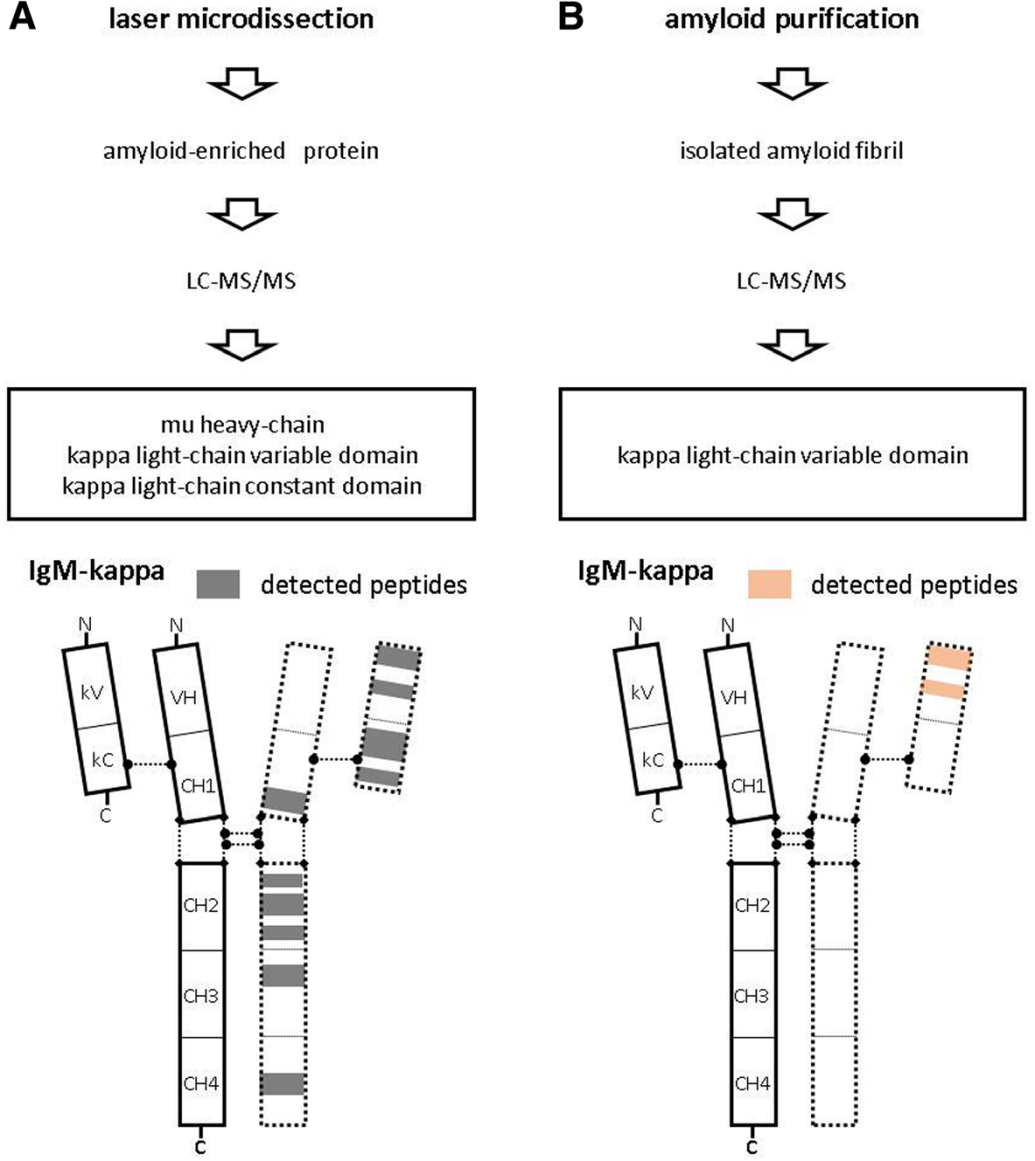
Schematic diagrams of the two-LC-MS/MS analyses. **a** LMD-LC-MS/MS identified mu heavy-chain and kappa light-chain. The identified peptide sequences were identical to amino acid position 79–89, 121–128, 132–150, 164–177, 244–238, and 377–391 of mu heavy-chain, position 1–18 and 47–55 of kappa light-chain variable domain, and position 41–75 and 83–99 of kappa light-chain constant domain. **b** LC-MS/MS of isolated amyloid fibrils only identified kappa light-chain. The identified peptide sequences were identical to amino acid position 1–18 and 47–55 of kappa light-chain variable domain

In order to confirm the monotypic IgM-kappa deposition and the diagnosis of AHL amyloidosis, we performed LMD-LC-MS/MS of the glomerular amyloid depositions. The LMD-LC-MS/MS identified mu heavy-chain and kappa light-chain (Fig. 2a) with mu heavy-chain predominance consistent with the diagnosis of IgM-kappa type renal AHL amyloidosis [3]. We next performed LC-MS/MS of renal amyloid fibrils isolated by the traditional amyloid purification method [7] to confirm that both the identified immunoglobulins compose amyloid fibrils. The additional LC-MS/MS only identified kappa light-chains without any heavy-chain component (Fig. 2b). All together, we eventually diagnosed the case as “kappa type renal AL amyloidosis with non-amyloid forming monoclonal IgM-kappa deposition”.

## Discussion

AHL amyloidosis is a disease entity where both the immunoglobulin light-chain and heavy-chain are composing amyloid fibrils, namely the concurrence of AL and AH amyloidosis [1, 4].

In AL amyloidosis, excess production of amyloidogenic light-chain lead to amyloid fibril formation [8]. In the present case, LMD-LC-MS/MS identified the peptide sequences of the kappa light-chain variable and constant domain (Fig. 2a), and the LC-MS/MS of isolated amyloid fibrils confirmed that variable domain were the component of the amyloid fibrils (Fig. 2b).

In contrast to AL amyloidosis, little is known about heavy-chain derived amyloid fibril formation. When reviewing the reported cases of AH amyloidosis (Table 1, [2, 3, 9–17]), it was presumable that amyloid fibrils are composed of the variable domains with or without truncated small constant domains of immunoglobulin heavy-chain [9, 10, 14–17]. In the present case, LMD-LC-MS/MS identified peptide sequences that were broadly distributed among the constant domain (CH1, CH2, CH3, and CH4 domains) of mu heavy-chain (Fig. 2a) that do not match the previously reported AH amyloidosis cases. Therefore, the LMD-LC-MS/MS results were suggestive that the identified mu heavy-chain was not composing amyloid fibrils, and the LC-MS/MS of isolated amyloid fibrils confirmed the speculation (Fig. 2b).

**Table 1:** Reported cases of renal immunoglobulin heavy-chain amyloidosis and amyloid responsible region of immunoglobulin heavy-chain

Age/Gen	Method for diagnosis	Sample preparation method	IEP	Immunostaining	Detail of amyloid protein	Size of amyloid protein	Structural abnormality of amyloid protein	Reported year	Ref
65/F	WB/AASA	Amyloid purification	IgG-κ	ND	V_H_ + Cγ_3_	22 kDa	V_H_ directly contiguous with CH3	1990	[9]
59/M	WB/AASA	Amyloid purification	IgG-κ	K(−), λ(−)	v_H_	11 kDa	V_H_ with truncated D segment	1994	[10]
64/M	Immunostaining		IgA-κ	IgA(+), κ(−), λ(−)	NA	NA	NA	2002	[11]
56/M	Immunostaining		IgM-λ	IgM(+), κ(−), λ(−)	NA	NA	NA	2003	[12]
53/F	Immunostaining		IgG-λ	IgG(+), κ(−), λ(+)	NA	NA	NA	2003	[13]
72/F	AASA	Amyloid purification	IgG-λ	IgG(−), κ(−), λ(−)	v_H_	11 kDa	truncated V_H_	2004	[14]
61/F	AASA	Amyloid purification	IgG-κ	IgG(−), κ(−), λ(−)	v_H_	11 kDa	V_H_ with truncated D segment	2006	[15]
67/M	LC-MS/MS	Amyloid purification	IgM-κ	K(−), λ(−)	v_H_	11 kDa	truncated V_H_	2008	[16]
50/M	LC-MS/MS	LMD	IgG-λ	ND	V_H_ + Cγ_3_	NA	NA	2010	[2]
36/M	LC-MS/MS	LMD	IgG-κ	IgG(+), IgA(+), IgM(+), κ(+), λ(+)	V_H_ + Cγ_3_	NA	NA	2010	[2]
ND	LC-MS/MS	LMD	IgA-λ	IgA(+), λ(+)	V_H_ + Cα_1_	NA	NA	2013	[3]
ND	LC-MS/MS	LMD	IgG-λ	IgG(+), λ(+)	Cγ_1_	NA	NA	2013	[3]
ND	LC-MS/MS	LMD	negative	IgG(+)	Cγ_1_, Cγ_4_	NA	NA	2013	[3]
ND	Immunostaining		IgG-λ	IgG_1_,(+)	NA	NA	NA	2013	[3]
70/F	LC-MS/MS	Amyloid purification	IgG-λ	IgG(−), κ(−), λ(−)	V_H_ + Cγ_1_	11 kDa	V_H_ with truncated CHI	2015	[17]

*IEP* immunoelectrophoresis, *Ref* reference, *WB* western blotting, *AASA* amino acid sequence analysis, *ND* not documented, *V*_*H*_ immunoglobulin heavy-chain variable domain, *C*γ_*3*_ gamma3 heavy-chain constant domain, *NA* not analyzed, *LC-MS/MS* liquid chromatography-tandem mass spectrometry, *LMD* laser micro dissection, *Cα* alpha heavy-chain constant domain

There are several hypotheses to explain the glomerular mu heavy-chain deposition. First, there are reported cases of AL amyloidosis combined with non-amyloid monoclonal immunoglobulin deposition diseases (MIDDs) [18]. However, in the present case, there were no histological findings to support the concurrence of non-amyloid MIDD [1]. Second, concurrence of AL amyloidosis and heavy-chain disease cannot be denied [19]. However, plasma electrophoresis did not revealed free heavy-chain and smears and flow cytometry of the bone marrow aspirate were consistent with the lymphoplasmacytic lymphoma without detection of IgM positive light-chain negative lymphoplasmacytoid lymphocytes [20], Therefore, we speculate that the identified mu heavy-chain was derived from monoclonal IgM-kappa that non-specifically co-deposited with amyloid fibrils [5], while the exact origin remains unknown.

This case documented “AL amyloidosis with non-amyloid forming monoclonal immunoglobulin deposition” using immunostaining and two distinct LC-MS/MS analyses. In fact, the existence of the present condition has been speculated upon the initial description of AHL amyloidosis [4]. The concern was in part because of the technical limitations of immunostaining and LMD-LC-MS/MS which do not differentiate amyloid fibrils from non-amyloid monoclonal immunoglobulin deposition [4, 5]. Moreover, because of the extremely low prevalence of AH amyloidosis [2–4, 21], it was questioned whether a single patient may develop both AL and AH amyloidosis [4]. On the other hand, co-deposition of non-amyloid immunoglobulin with amyloid fibrils is a well-described phenomenon [4–6, 22]. Therefore, we speculate that the present condition might not be so rare and might be more prevalent than “true” AHL amyloidosis.

## Conclusion

We demonstrated “AL amyloidosis with non-amyloid forming monoclonal Ig deposition” disguised as AHL amyloidosis. The confident exclusion of the present condition should be required to diagnose AHL amyloidosis.

### Concise method for the two LC-MS/MS analyses

LMD [2, 3, 18]: Formalin-fixed paraffin-embedded sections were stained with congo-red dye, and the positive areas were extracted using an LMD system (LMD 7000; Leica Microsystems Inc., Tokyo, Japan). The extraction was solubilised in 10 mM Tris/1 mM EDTA/0.002% Zwittergent buffer and digested overnight with trypsin.

Amyloid purification [7, 14–17]: Fresh frozen renal tissue was homogenised in Tris-buffered saline, after which it was centrifuged and the supernatant was decanted. The procedure was repeated twice. The resultant pellet was solubilised in 6 M guanidine/0.5 M Tris-buffered saline, after which it was centrifuged and the supernatant was dialysed against distilled water. The sample was solubilised in gel loading buffer containing 5% 2-mercaptoethanol and subjected to sodium dodecyl sulfate polyacrylamide gel electrophoresis. The entire electrophoresed sample was excited and digested overnight with trypsin.

The samples were analysed by LC-MS/MS (Nano LC DiNa; KYA Technologies Co., Tokyo, Japan; and QExactive; Thermo Fisher Scientific Inc., Waltham, MA).

## Abbreviations

AH amyloidosis: Immunoglobulin heavy-chain amyloidosis
AHL amyloidosis: Immunoglobulin heavy-and-light-chain amyloidosis
AL amyloidosis: Immunoglobulin light-chain amyloidosis
Ig: Immunoglobulin
LC-MS/MS: Liquid chromatography-tandem mass spectrometry
LMD: Laser microdissection
UPCR: Urinary protein-to-creatinine ratio
